# Harnessing dairy wastewater to cultivate *Scenedesmus* sp. for biofertilizer applications in *Phaseolus vulgaris* L.: a sustainable agro-biotechnological approach

**DOI:** 10.3389/fpls.2025.1568057

**Published:** 2025-05-01

**Authors:** Xavier Álvarez-Montero, Ingrid Mercado-Reyes, Wiliam Castillo-Chamba, Efrén Santos-Ordóñez

**Affiliations:** ^1^ Universidad de las Fuerzas Armadas-ESPE, Sede Santo Domingo, Departamento de Ciencias de la Vida y Agricultura, Santo Domingo, Ecuador; ^2^ Laboratorio de Inocuidad Alimentaria, Escuela de Medicina Veterinaria, Universidad Nacional Andrés Bello (UNAB), Santiago, Chile; ^3^ Doctorado en Biotecnología, Facultad de Ciencias de la Vida, República, Universidad Nacional Andrés Bello (UNAB), Santiago, Chile; ^4^ Centro de Investigaciones Biotecnológicas del Ecuador, ESPOL Polytechnic University, Escuela Superior Politécnica del Litoral (ESPOL), Guayaquil, Ecuador; ^5^ Facultad de Ciencias de la Vida, ESPOL Polytechnic University, ESPOL, Escuela Superior Politécnica del Litoral (ESPOL), Guayaquil, Ecuador

**Keywords:** *Scenedesmus* sp., dairy wastewater, bio-stimulants, bioremediation, bean

## Abstract

Transforming traditional linear production into sustainable circular processes is crucial, and integrating microalgae biomass production with wastewater recycling is a promising approach. This study addresses key challenges using dairy industry effluents as a nutrient-rich medium, achieving high biomass productivity and protein content with *Scenedesmus* sp. grown in an 80% wastewater-based medium. Impressive nutrient removal efficiencies were recorded for TN (79.24%) and PO_4_^-3^ (77.14%). The proposed culture medium achieved maximum productivity of 0.22 ± 0.05 g L^−1^ day^−1^ and a high protein concentration of 384.38 ± 34.06 mg g^−1^, demonstrating the medium’s efficiency in promoting substantial biomass and nutritional quality. The application of *Scenedesmus* sp. biomass in treatment T_1_ (extract) and T_2_ (culture) in *Phaseolus vulgaris* significantly improved soil quality, increasing the concentration of organic matter (SOM), nitrates, phosphates, and microbial activity. Additionally, T_1_ promoted the vegetative and reproductive development of *P. vulgaris*, as reflected in a germination index of 305.81%, an average height of 49.52 cm, higher leaf density, a greater number of floral buds, and enhanced floral development. These results demonstrate the bio-stimulatory potential of biomass and its role in practical bioremediation, highlighting the environmental and agricultural benefits of this innovative approach.

## 1 Introduction

The food industry, a key pillar in the food supply chain, is one of the largest water consumers and generators of residual effluents. It is estimated that the dairy industry, which is projected to grow rapidly by 22% by 2030, consumes between 0.2 and 11 liters of water per liter of processed dairy products. However, in some cases, this consumption could reach 60 liters per kilogram of processed milk. Between 0.5 and 20.5 liters of wastewater are generated depending on the composition and variety of the final products, which typically have a high biological oxygen demand (BOD) and chemical oxygen demand (COD) and contain complex organic and inorganic compounds. These compounds include acid whey, lactose, fatty substances, proteins, salts, and various cleaning chemicals. Additionally, almost 2.5 times the volume of processed milk is generated as wastewater through washing or spillage, making it harmful to ecosystems if not adequately treated beforehand (Álvarez et al., 2020; Pant et al., 2023; Sharma et al., 2024).

Due to the concentration of organic load and nutrients in this type of wastewater, biotreatments are a more practical option than other methods. They provide a medium rich in compounds that microorganisms can assimilate, representing a profitable alternative for obtaining biomass with multiple applications and for the purification of water masses. A commonly used microbial group in biological treatment is microalgae, which serve as potential platforms for reducing pollutants, nutrients, BOD, COD, heavy metals, and industrial atmospheric CO_2_ (Do et al., 2019).

Phycoremediation, based on microalgal metabolism and their adaptability to different substrates, represents an advantageous system for reducing the cost of nutrients and freshwater consumption in producing biomass with high added value. This approach favors scale production processes while protecting ecosystems from the risk of eutrophication and excessive accumulation of CO_2_ in the atmosphere (Chai et al., 2021; Geremia et al., 2021). The biomass generated through these systems has a variety of sustainable applications, including the production of renewable fuels, biostimulants, biofertilizers, biohydrogen, bio-alcohols, bio-hydrocarbons, or commercial products based on bioactive compounds such as proteins, pigments, antioxidants, fatty acids, amino acids (Do et al., 2019; Wollmann et al., 2019). This viability extends to the agriculture, animal feed, and bioenergy sectors.

Dairy wastewater has been explored as a potential substrate for producing microalgae biomass. Brar et al. (2019) conducted a study where they cultivated *Chlorella pyrenoidosa*, *Anabaena ambigua*, and *Scenedesmus abundans* in dairy wastewater at 75% (v/v). They observed that the strains tended to increase the dry weight while minimizing external nutrient requirements. Additionally, high percentages of assimilation of BOD, COD, nitrates, and phosphates (> 56%) were achieved. The biomass produced can be utilized to obtain bioenergy by estimating the theoretical potential of the species to produce biogas, as demonstrated by Álvarez et al. (2020). They used biomass of *Arthrospira platensis* grown in dairy wastewater as a substrate in mesophilic anaerobic co-digestion with cattle manure. It was found that the digestion of these co-substrates allows for a 2.5-fold increase in methane yield compared to the control (cattle manure), indicating that the microalgal biomass produced in dairy effluents is rich in protein, lipid, and carbohydrate compounds, which supply energy and nutrients in the fermentation process.

The composition of the algal biomass obtained will vary depending on the specific characteristic of each effluent, which directly influences the metabolism of the strain used. For instance, Mercado et al. (2020) demonstrated that dairy wastewater induces a notable production of energy compounds and cellular morphological changes. The authors cultivated the strain *Scenedesmus* sp. in dairy wastewater with high mercury concentrations (0.443 mM) within the maximum permissible limits. They found that lipid synthesis increases up to 51% of the total dry weight, representing a 45% increase in lipid content compared to the control. Additionally, the substrate changed cell size, increasing 1.8 and 1.5 times the cell area and volume, respectively. This change was mainly attributed to the redirection of carbon metabolic flux towards generating reserve compounds and enhanced absorption of nutrients and light energy.

Due to their exceptional metabolic flexibility, Microalgae have demonstrated a remarkable adaptive capacity to various residual effluents. However, this adaptability relies on the specific resistance of the strain employed. According to Choi et al. (2018), strains isolated directly from dairy effluents exhibit enhanced growth and lipid production due to their resistance to increased turbidity, COD, nutrients, and trace elements. This resilience allows for the utilization of residuals without any modification, thereby enhancing the profitability of the system. The genera *Scenedesmus*, *Chlorella*, *Chlamydomonas*, and *Euglena* are among the most commonly used in wastewater treatment due to their high tolerance to the compounds present in these effluents, which makes them robust strains with great potential for such applications (Abdel-Raouf et al., 2012).

The selection and identification of robust and productive strains pose a significant challenge when coupled with wastewater treatment processes. It is important to note that microalgae are highly diverse microorganisms, forming a polyphyletic group. This diversity renders traditional taxonomic classification and identification processes (phenotypic characterization) impractical due to subjectivity criteria (Ancona-Canché et al., 2017). Currently, molecular techniques based on the extraction and purification of cellular DNA are employed. These techniques identify specific regions of the strains by utilizing PCR to amplify the 16S rRNA region using universal primers, thus ensuring greater robustness in strain identification.

Furthermore, the composition of the produced biomass will determine its final applications, such as its use as animal feed or biofertilizers, which are among the most promising options; however, it is essential to note that utilizing the biomass in applications related to human consumption is prohibited due to safety reasons. One of the main potential applications is in agriculture, where microalgae contribute nutrients to the soil and produce molecules like phytohormones and amino acids that promote crop development (Gouveia et al., 1997; Miranda et al., 2024).

Dairy wastewater is an important source of nutrients that exacerbates sanitary and environmental issues. However, it could represent a sustainable alternative for microalgae cultivation with industrial applications in areas such as agriculture, energy, and animal nutrition. Therefore, this study supports the use of such effluents to cultivate native strains from Ecuador in biotreatment systems, analyzing growth, productivity, biochemical composition, and nutrient assimilation efficiency. Subsequently, molecular techniques based on the amplification of the ITS molecular marker are proposed to identify the organism at the phylogenetic level and obtain more reliable results, given its high specificity in eukaryotic species. To give an application to the produced biomass, the study will explore its use in legume cultivation, evaluating potential bio-stimulant effects. Essentially, this research is aimed at discovering sustainable processes that have not been previously addressed with native strains, providing a significant opportunity for low-cost industrial processes.

## 2 Materials and methods

### 2.1 Microalgal strain used in this study

The microalga utilized in this study originates from the microalgae and cyanobacteria strain bank of the Microbial Biotechnology Laboratory (Lab-Biotem S.A.) in Guayaquil, Guayas, Ecuador. It was isolated from the Jauneche Reserve - Pedro Franco Dávila Research Station (1°14’50.2” S; 79°39’34.5” W) and is coded as LBM-0020. This strain is stored in the standard medium BG11 pH 7.8 at a temperature of 22 ± 2°C and exposed to a light intensity of 30 µmol photon m^-2^ s^-1^ under a circadian regime of light radiation (12 hours light: 12 hours dark).

### 2.2 DNA barcoding and phylogenetic analysis

DNA extraction was conducted following a modified protocol based on Morin et al. (2010). Initially, 5 mL of cell culture underwent centrifugation at 1200 rpm for 20 min. Subsequently, the precipitate was treated with 500 μL of TES lysis buffer (100 mM Tris; pH 8.0; 10 mM EDTA; and 2% SDS) and subjected to sonication at 20 kHz at 4°C for 20 min. The sonicated sample was then transferred to a 1.5 mL Eppendorf tube, then 12.56 μL of Proteinase K was added and incubated at 37°C for 30 minutes. Next, 140 μL of 5 M NaCl and 1/10 volume of 10% CTAB were added to the tube and then incubated at 65°C for 10 min. Subsequently, one volume of chloroform: isoamyl alcohol (24:1) was added, and the tube was placed on ice for 5 minutes before centrifugation at 14,000 rpm for 10 min at 4°C. The resulting supernatant was carefully transferred to a new 2 mL Eppendorf tube. Following this, 225 μL of 5 M ammonium acetate was added to the supernatant, left on ice for 5 min, and then centrifuged at 14,000 rpm for 5 min at 4°C. To precipitate the DNA, 0.5 volumes of isopropanol were added to the supernatant, which was then allowed to react at -20°C overnight. Finally, the tube was centrifuged at 14,000 rpm for 20 min at 4°C, yielding a DNA pellet. The pellet was washed with 1 mL of cold 70% ethanol and centrifuged at 14,000 rpm for 5 min at 4°C. Subsequently, the pellet was air-dried in a laminar flow chamber for 10 to 15 min and then resuspended in 200 μL of ultra-pure water for further analysis.

Following the modified protocol of Ancona-Canché et al. (2017), DNA amplification was carried out in triplicate PCR reactions, each prepared in a final volume of 10 μL. Each primer, ITS1 (5´- TCC GTA GGT GAA CCT GCG G -3´) and ITS4 (5´-TCC TCC GCT TAT TGA TAT GC-3´) was used at a final concentration of 500 nM.

The PCR reaction mixtures consisted of 5 µL of GoTaq^®^ Green Master Mix (1x), 2 µL of H_2_O, and 2 µL of DNA template. These components were added to each PCR reaction tube. Subsequently, the tubes were placed in a thermal cycler programmed to undergo thermal cycling as follows: initial denaturation at 95°C for 5 minutes, followed by 35 cycles of denaturation at 95°C for 30 seconds, annealing at 55°C for 1 minute, extension at 72°C for 2 minutes, and a final extension step at 72°C for 5 minutes. Following PCR amplification, the products were subjected to commercial sequencing (Macrogen, Rockville, MD, USA) after purification.

The analysis of all the sequences was conducted using Mega X software. Following alignment, the sequences were trimmed at both ends to obtain a consensus sequence, which was then uploaded to GenBank. Subsequently, the sequence was blasted for similarity. For phylogenetic analysis, different accessions were employed after alignment with MUSCLE from MEGA X. The maximum likelihood method was utilized for phylogenetic reconstruction, with a bootstrap test of 1000 replicates. The best model for the analysis was determined by MEGA X (Kumar and Chaurasia, 2018).

### 2.3 Culture medium and experimental setup

The wastewater utilized as an alternative culture medium originates from the distribution channel of a dairy industry (*DIWW*) specializing in dairy products and gourmet cheeses. It possesses the following initial chemical characteristics: pH: 5.5, Total nitrogen (TN): 2.22 mM, Phosphate (PO_4_^-3^): 0.20 mM, and Chemical Oxygen Demand (COD): 3500 mg L^-1^.

The growth of microalgae was assessed using gradual concentrations of 10 to 80% of wastewater to minimize adaptation time at the onset of cultures. For the test control, the standard medium BG11 was employed, comprising the following components: NaNO_3_ 17.65 mM; K_2_HPO_4_ 0.23 mM; MgSO_4_·7H_2_O 0.30 mM; CaCl_2_·2H_2_O 0.25 mM; Na_2_CO_3_ 0.38 mM; HOC(COOH)(CH_2_COOH)_2_ H_2_O 0.03 mM, C_6_H_8_O_7_·xFe_3_NH_3_ 0.03 mM; 0.003mM EDTA; and by elements: H_3_BO_3_ 0.046 mM; MnCl_2_·4H_2_O 0.009 mM; ZnSO_4_·7H_2_O 0.0008 mM; Na_2_MoO_4_·2H_2_O 0.0016 mM; CuSO_4_·5H_2_O 0.0003 mM; Co(NO_3_)_2_·6H_2_O 0.0002 (Sigma-Aldrich, St. Louis, MO, USA) (Allen, 1968).

The treatments involved the use of dairy industry water (DIWW) at an 80% concentration and 20% seawater (DIWW 80% + SW 20%), supplemented with Bayfolan^®^ fertilizer (BAYER S.A., Quito, EC) at a dose of 2.5 mL L^−1^. Each culture was conducted in quadruplicate.

The seawater was collected from the Palmar commune, in the province of Santa Elena, Ecuador (-2.025388, -80.736178), and had a salinity of 33‰ with the following composition: Cl: 18,900 ppm, Na: 10,850 ppm, SO_4_²-: 2,750 ppm, Mg: 1,370 ppm, S: 880 ppm, Ca: 405 ppm, K: 380 ppm, HCO_3_-: 140 ppm, Br: 65.9 ppm, H_3_BO_3_: 25 ppm, F: 12 ppm, Sr: 8.5 ppm, B: 4.4 ppm, Si: 2.8 ppm; also, the composition of Bayfolan^®^ fertilizer, detailed in **Table 1**, resulted in a final concentration of 18.26 mM total nitrogen and 1.59 mM PO_4_^3−^.

**Table 1:** Composition of the liquid foliar fertilizer Bayfolan^®^ 11-8-6 (N-P-K).

Elements/compounds	Concentration (M)
Total nitrogen	6.43 M
PO_4_^-3^	0.63 M
K_2_O	0.74 M
Fe	3.40 mM
Mn	2.91 mM
B	9.34 mM
Mo	0.10 mM

### 2.4 Photobioreactor setup and culture conditions

The microalgae was cultivated in batch in four bubble-column photobioreactors with a total volume of 300 mL and a culture volume of 200 mL. The initial cell density was set to 1.5 x 10^7^ cell mL^-1^. The reactors were illuminated artificially with a photoperiod of 12 hours of light and 12 hours of darkness, providing an irradiance of 100 µmol photon m^-2^ s^-1^ through white light LED lamps. The culture temperature was maintained at 24 ± 2°C. Mixing was facilitated by a continuous airflow of 0.1 v/v/min, which also helped keep the dissolved oxygen concentration in the 100 ± 10% saturation range. To regulate the pH at 7.0 ± 0.3, on-demand injections of CO_2_ were administered.

The photoreactors were sampled daily to determine biomass and growth rate. Additionally, biochemical analyses were carried out at the end of the batch culture. Various analytical methods were employed to determine the growth parameters and the biochemical composition of the biomass. This included daily cell concentration measurements, dry biomass, and quantifying carbohydrates, proteins, and lipids after the trial. The percentage uptake of total nitrogen and phosphorus was also assessed to evaluate nutrient removal efficiency from the medium.

### 2.5 Analytical methods for evaluating growth, biomass composition, and nutrient removal efficiency

The cell concentration was determined daily following the method outlined by Guillard and Sieracki (2005). This involved using a Neubauer hemocytometer chamber, with 10 uL of homogeneous culture added to the chamber. The cells in the central area squares were then counted using the Equation 1:


(1) $$C\;=N\;\times \;10^{4}\;x\;f$$

Where:

*C* is the cell concentration (cell mL^-1^),

*N* is the average number of cells counted in 1 mm (0.1 μL),

and *f* is the dilution factor.

The dry weight of the biomass (g L^-1^) was determined using the methodology proposed by Zhu and Lee (1997). The process began by drying the glass fiber filters (Whatman^®^ GF/F with a diameter of 47 mm and a pore diameter of 0.7 µm) at 90°C in an oven for 24h to prevent moisture absorption. Subsequently, the dried filters were stored in a desiccator with silicon oxide (SiO_2_), and the weight of the dried filters was recorded as initial weight using an analytical balance. Next, 5 mL of homogeneous culture was extracted from each test culture, and 5 mL of 0.5 M ammonium formate (HCO_2_NH_4_) solution was added to eliminate the salts present in the culture media. Each sample was filtered and returned to the oven at 72°C for 24 hours. After drying, the filters were weighed again using the analytical balance. The total dry weight was determined by calculating the difference between the final weight of the filter with the dry biomass and the initial weight of the dry filter. This process provided the dry weight of the biomass in grams per liter (g L^-1^) of culture.

Based on the biomass production data, the biomass productivity (Pb) was determined using the Equation 2 proposed by Mohd Apandi et al. (2021):


(2) $$Pb\;=\;\frac{X_{2}-\;X_{1}}{t_{2}\;-\;t_{1}}$$

Where:

*Pb* is biomass productivity.

*X_1_* and *X_2_* are the biomass concentrations at times *t_1_* and *t_2_*, respectively.

*t_1_* and *t_2_* are the corresponding times.

These equations allow for calculating biomass productivity based on the changes in cell density or biomass concentration over time, providing valuable insights into the growth dynamics of microalgae cultures.

The concentration of carbohydrates in the biomass was determined using the phenol-sulfuric method proposed by Dubois et al. (1956), involving three steps.

First, 1 mL of sample from each culture was collected and centrifuged. The resulting pellet was resuspended in 1 mL of 1N NaOH, followed by homogenization in a vortex and sonication at 20 kHz for 20 min at 4°C. After sonication, the samples were centrifuged at 8000 g for 15 minutes at 4°C. Second, a dilution (1:10) was prepared in triplicate test tubes. Each test tube contained 100 μL of the supernatant from each sample, to which 900 μL distilled H_2_O was added. Subsequently, 25 μL of 80% phenol and 2.5 mL of concentrated H_2_SO_4_ were added to each tube. The contents were mixed thoroughly using a vortex. Finally, the samples were allowed to cool for 30 min, after which the carbohydrate concentration was measured using UV-VIS spectrophotometry at 485 nm, using glucose as the reference standard.

The protein fraction of the biomass was determined using the method described by Lowry et al. (1951), with modification by Herbert et al. (1971). The procedure involved several steps: 10 mL of culture was centrifuged, and the resulting pellet was treated with 2 mL of 1N NaOH. The sample was then sonicated at 20 kHz and 4°C for 15 minutes, followed by incubation in a thermostatic bath at 95°C for one hour to denature the proteins. After cooling the samples to room temperature, they were centrifuged at 3800 g for 15 min to remove any insoluble debris. In triplicate test tubes, 100 μL of the supernatant from each sample, 400 μL of distilled water, 300 μL of 1N NaOH, and 2 mL of saturated Cu^+2^ tartrate solution were added. The mixtures were allowed to react for 10 minutes, adding 400 µL of diluted Folin-Ciocalteu (1:1 v/v). The contentsh of the test tubes were mixed thoroughly using a vortex and allowed to react for an additional 30 minutes. Finally, the protein concentrations of each sample were determined by spectrophotometry at an absorbance of 750 nm. The data obtained are quantified by interpolating the bovine serum albumin (BSA) standard curve.

The concentration of biomass lipids was determined using the Bligh and Dyer method (Bligh and Dyer, 1959), followed by the Marsh and Weinstein method (Marsh and Weinstein, 1966). The procedure involved several steps: 15 mL of biomass of each culture was dried to extract 5 mg of dry biomass. Three mL of methanol and 1.5 mL of chloroform were added to the dried biomass. The mixture was homogenized and sonicated at 20 kHz and 4°C for 20 min, followed by incubation in a thermostatic bath at 50°C for 30 minutes. After cooling, the samples were centrifuged at 3900 rpm for 10 minutes to separate the supernatant. 1.5 mL of chloroform and 1.5 mL of distilled water were added to the supernatant. The supernatant was removed, and 0.5 mL of acetone was added to the precipitate to eliminate the remaining compounds. The solution was evaporated under a constant nitrogen flow until 1 mL of chloroform remained. Following this, the Marsh and Weinstein method (Marsh and Weinstein, 1966) was applied. Specifically, 2.5 mL of concentrated H_2_SO_4_ was added to 100 µL of the sample resuspended in chloroform in triplicate. Carbonization was then carried out at 200**°**C for 15 min. After cooling, 3 mL of distilled water was added to each tube to determine the lipid concentration later by spectrophotometry at 375 nm, using palmitic acid as the standard. The analyses for biochemical characterization were performed in triplicate for each photobioreactor.

After the trial, 10 mL of sample was extracted from each culture for analysis of total nitrogen and total phosphorous concentration. The total nitrogen concentration was determined using an adapted version of the persulfate digestion method (D’Elia et al., 1977). This method involves oxidizing the organic nitrogen compounds to nitrate using persulfate, followed by spectrophotometric measurement of the resulting nitrate concentration. On the other hand, the total phosphorus concentration was determined using the vanadate-molybdate method (Tandon et al., 1968). In this method, phosphorus reacts with ammonium molybdate and potassium antimonyl tartrate in an acidic medium to form a complex, which is then reduced to a blue color by ascorbic acid. The intensity of the blue color is proportional to the concentration of phosphorus, allowing for spectrophotometric measurement.

The concentrations were measured in the Lovibond^®^ MD600/MaxiDirect photometry equipment following standard protocols. These methods provide accurate and reliable measurements of total nitrogen and total phosphorus concentrations in the samples, enabling the assessment of nutrient removal efficiency from the wastewater by the microalgae cultures.

To calculate the nutrient removal efficiency (Re), the percentage difference between the initial (Ci) and final (Cf) concentrations of each nutrient in the culture can be determined using the Equation 3:


(3) $$Re\;=100\;-\;\frac{C_{f}\;\times 100}{C_{i}}$$

Where:

*Re* is the nutrient removal efficient (in percentage).

*Cf* is the final concentration of the nutrient.

*Ci* is the initial concentration of the nutrients.

This equation measures the percentage reduction in nutrient concentration achieved by the microalgae culture throughout the trial, indicating nutrient removal efficiency from the wastewater.

### 2.6 Biostimulation with *Scenedesmus* sp.: impact on *Phaseolus vulgaris* L. growth and soil quality

The potential of *Scenedesmus* sp. as a biofertilizer/biostimulant was evaluated in crops of *Phaseolus vulgaris* L. (common bean) under various application conditions. The application of cellular extract and live culture was explored, as detailed in **Table 2**.

**Table 2:** Treatments applied in the cultivation of *Phaseolus vulgaris* L.

Treatment	Description
C-	Water, negative control
C+	Commercial foliar fertilizer, positive control
T_1_	*Scenedesmus* sp. cellular extract (4.4 ± 0.1 x 10^7^ cell mL^-1^)
T_2_	Live culture of *Scenedesmus* sp. (4.4 ± 0.1 x 10^7^ cell mL^-1^)

Control negative was used using only water, while the positive control was Evergreen^®^ (ExcelAg, Florida, MIA, USA) foliar fertilizer with the following composition (w/v): 7.77% N, 9.98% P_2_O_5_, 07.00% K, 0.05% Fe, 0.04% Mg, 0.02% B, 0.01% Cu, 0.01% Mn, 0.01% Zn, 0.59% humic acid, Mo 0.0003%, 40 ppm auxins, 40 ppm gibberellins, 90 ppm cytokinins, 750 ppb choline, pantothenic acid 12 ppb, folic acid one ppb, thiamine 100 ppb, niacin 85 ppb, nicotinamide two ppb, riboflavin 100 ppb.

Regarding the biomass of the evaluated microalgae, *Scenedesmus* sp. was cultivated under the conditions mentioned above. For treatments containing *Scenedesmus* sp. cellular extract, ultrasound pulses were used (Portable Ultrasonic Cell Disruptor, UCD-P01, Biobase^©^) at 20 kHz, 20 minutes, 4°C, for cell rupture and release of intracellular components of the microalgae.

**Figure 1:**
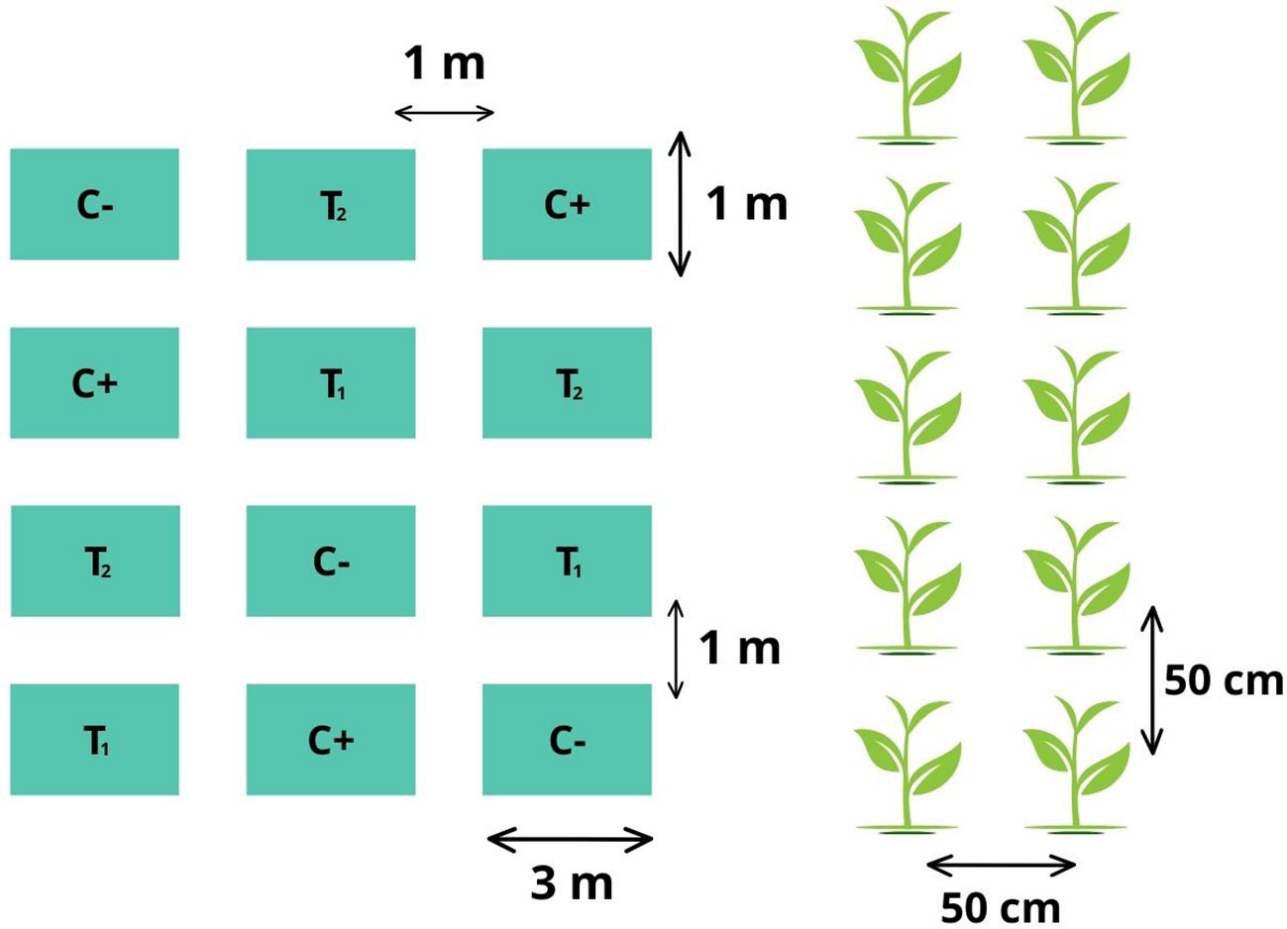
Scheme of the experimental design of the trial with its respective replications.

A completely randomized (CRD) experimental design was used, employing two controls and two treatments, with three replications each. The detail of the experimental units is described below: 77 m^2^ total area of the experiment, 12 experimental units, 3 m^2^ area of each plot, 1 m spacing between plots, ten seed holes plots, 50 cm spacing between seed holes, 40 cm spacing between plants, three seeds per seed hole, a total of 360 plants in the trial (**Figure 1**).

In each assay, 100 common beans (*P. vulgaris*, L.) seeds were analyzed, 25 seeds × 4 sterilized Petri dishes, placed on Whatman No. 5 filter papers. They were then treated with 2 mL of sterile distilled water (control -) and the same volume for the control + and the treatments; subsequently, they were sealed with parafilm and placed for 72 hours in a growth chamber at 25°C model LDC700BXPRO DAY-ILD200LED (FDM Environment Makers, Rome, ITA) (Navarro-López et al., 2020; Xie et al., 2022). The germination index was determined by the Equation 4 (Zucconi et al., 1981). The length of germinated bean seeds was measured with an electronic digital caliper (ADORIC^®^ 150 mm, Adoric Technologies LTD, Tel Aviv, IL):


(4) $$GI\;(\%)=\;\frac{G\;\times L}{G_{W\;}\;\times \;L_{W}}\;\times 100$$

Where:

*GI* is the germination index (in percentage).

*G* is the number of germinated seeds for the C+ and both treatments.

*L* is the length of the germinated seeds in the case of C+ and both treatments.

*G_W_* is the number of germinated seeds in the case of the negative control.

*L_W_* is the length of the germinated seeds in the case of the negative control.

In the initial phases, on the fourth day after planting, irrigation was carried out at a rate of 1 L per plot, and during the following five weeks, a manual pump was used at 120 mL m^-3^. The following physicochemical and microbiological analyses were carried out before delimiting each plot and at the end of the trial: pH, cell density in CFU g^-1^, NO_3_- by the phenol sulfonic acid method (Bremner & Keeney) (Bremner and Keeny, 1966), organic carbon by the Walkley & Black method (Walkley and Black, 1934) for subsequent calculation of Soil Organic Matter (SOM) content, and PO_4_^-3^ by the vanadate-molybdate method (Tandon et al., 1968).

By determining CFU g^-1^ of soil, the method of spreading sowing on plates was used; one g of soil was weighed and transferred to a test tube with 9 mL of 0.1% peptone solution. It was then mixed in a vortex for two minutes. Successive decimal dilutions (10^−1^, 10^−2^, 10^−3^) were made in new tubes with 9 mL of 0.1% peptone solution, using a sterile pipette, 100 μL of each dilution was inoculated in sterile Petri dishes with Plate Count Agar (PCA) medium, spreading the inoculum over the surface of the medium using a sterile Drigalski spatula. The procedure was repeated for each dilution in triplicate. The incubation was carried out for 24-48 h at 35°C. Once the colonies were visible, the plates containing between 30 and 300 CFU were counted. The number of CFU g^-1^ of soil was calculated using the Equation 5 (Laboratory for Environmental Pathogens Research Department of Environmental Sciences University of Toledo):


(5) CFU g−1 of soil = (Number of colonies × dilution factor)Volume sown (mL)Weight of the sample (g)

After planting, phenological parameters corresponding to germination index (gibberellin-like effects), growth (stem height), and number of leaves (auxin-like effects), pre-flowering and flowering (cytokinin-like effects) were evaluated through weekly monitoring for each treatment, as detailed in **Table 3**.

**Table 3:** Phenological parameters evaluated in the cultivation of *Phaseolus vulgaris* L.

Phenological stage	Germination	Growth (stem height)	Number of leaves	Pre-flowering	Flowering
Weeks after planting	0-1	1-5	1-5	4-6	4-6
Variable	% Germination	Height (cm).	Number of leaves.	Number of flower buds	Number of flowers

Plant height was analyzed from the sixth day post-planting with weekly measurements considering the dimension from the base of the stem to the apex; the number of leaves was determined by weekly counting considering developed and open leaves; in pre-flowering, the first flower buds in each treatment were recorded; and, for flowering, floral abundance was recorded when each treatment reached 50% of developed flowers in the axillary zone of the stem.

### 2.7 Reproducibility of the results and statistical analyses

The experimental design ensures the reproducibility of the results. For the cultivation of *Scenedesmus* sp. and the evaluation of its growth in the formulated medium (80% DIWW + 20% seawater), four photobioreactors per treatment were used, totaling eight experimental units. The analyses were performed in triplicate for each photobioreactor.

For the biomass application in *P. vulgaris*, four treatments (C+, C-, T_1_, T_2_) were evaluated, each with three replicates, totaling twelve experimental units. The germination index was evaluated with four replicates per treatment, totaling sixteen experimental units. All analyses were performed in triplicate for each experimental unit.

Prism version 10.4.1 (GraphPad Software, Inc^®^, CA, USA) was used for statistical analyses. A typical distribution analysis was performed using the Lilliefors variant of the nonparametric Kolmogorov-Smirnov test on the treatments to determine the parametric test. A one-way double parametric ANOVA and a Tukey *post hoc* test were performed.

## 3 Results

Microalgae are microorganisms that, due to their composition, growth patterns, or adaptability to various cultural sources, play an essential role in different areas of industrial production. To develop adequate processes based on these microorganisms, specific microalgae strains and culture media must be selected, and reliable applications of the produced biomass must be identified.

### 3.1 Identification of selected strains

**Figure 2:**
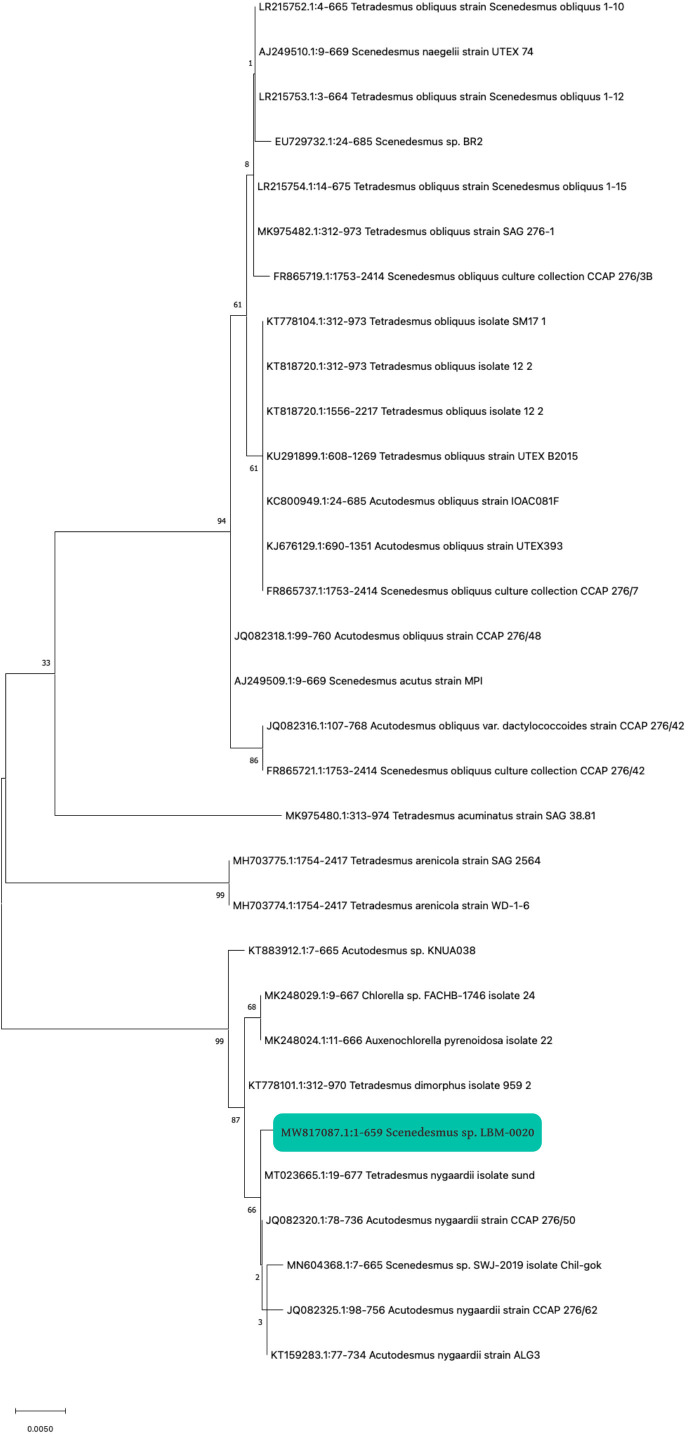
Phylogenetic tree of the isolate *Scenedesmus* sp. with code LBM-0020. The *Scenedesmus* sp. isolate is indicated with a green square.

The strain to be produced was selected based on previous experience confirming the reliability of this strain in different wastewater types. This strain was isolated from a water body habitually contaminated with different effluents. The first step to using the selected strain was identification by DNA analysis. PCR amplification was obtained using the primers ITS1/ITS4. After sequencing the PCR products, a consensus sequence was generated from three technical replicates using Mega X. The unique sequence contains 659 bp in length, harboring a partial sequence of the small and large subunit of ribosomal RNA, and a complete sequence for ITS1, the 5.8s rRNA gene, and ITS2. The sequence was deposited in GenBank with accession code MW817087 (https://www.ncbi.nlm.nih.gov/nuccore/MW817087). A BLAST analysis was performed in the GenBank database, yielding a similarity range of 99.7–99.85% with sequences from various isolates of the Scenedesmaceae family. The phylogenetic tree, constructed using MEGA X software with the “Maximum Likelihood” nucleotide substitution method (K2 + G model), shows that several accessions of the genera *Scenedesmus* and *Tetradesmus* form a clade with the LBM-0020 isolate (**Figure 2**), highlighting the taxonomic complexity of these genera (Fučíková et al., 2014; Wynne and Hallan, 2015). Although the phylogeny of the isolate suggests a closer relationship with *Tetradesmus*, it was classified as *Scenedesmus* sp. for the following reasons:

The initial identification of the isolate was based on key morphological criteria, including cell shape, colony arrangement, and the presence of spines or ridges, characteristics consistent with the genus *Scenedesmus*. Although phylogenetic analysis suggests a closer relationship with *Tetradesmus*, phenotypic traits remain a relevant and valuable criterion, particularly when genetic analyses yield inconclusive results (Hegewald, 2000; Krienitz et al., 2011). Recent taxonomic studies have shown that *Tetradesmus* and *Scenedesmus* are closely related, and some authors have proposed that *Tetradesmus* should be considered a synonym of *Scenedesmus* due to the overlapping of their genetic and morphological characteristics (Fučíková et al., 2014; Wynne and Hallan, 2015). Therefore, the distinction between these genera remains a matter of debate within the scientific community. Several previous studies have classified strains phylogenetically close to *Tetradesmus* as *Scenedesmus* due to the lack of taxonomic consensus and the prioritization of morphological criteria (Hegewald, 2000; Krienitz et al., 2011). We have chosen to follow this convention to maintain consistency with the existing literature. Nonetheless, it has also been proposed that the isolate could be identified as *Scenedesmus/Tetradesmus* to acknowledge its phylogenetic similarity to *Tetradesmus* while maintaining its initial morphological classification.

### 3.2 Biomass growth

**Figure 3:**
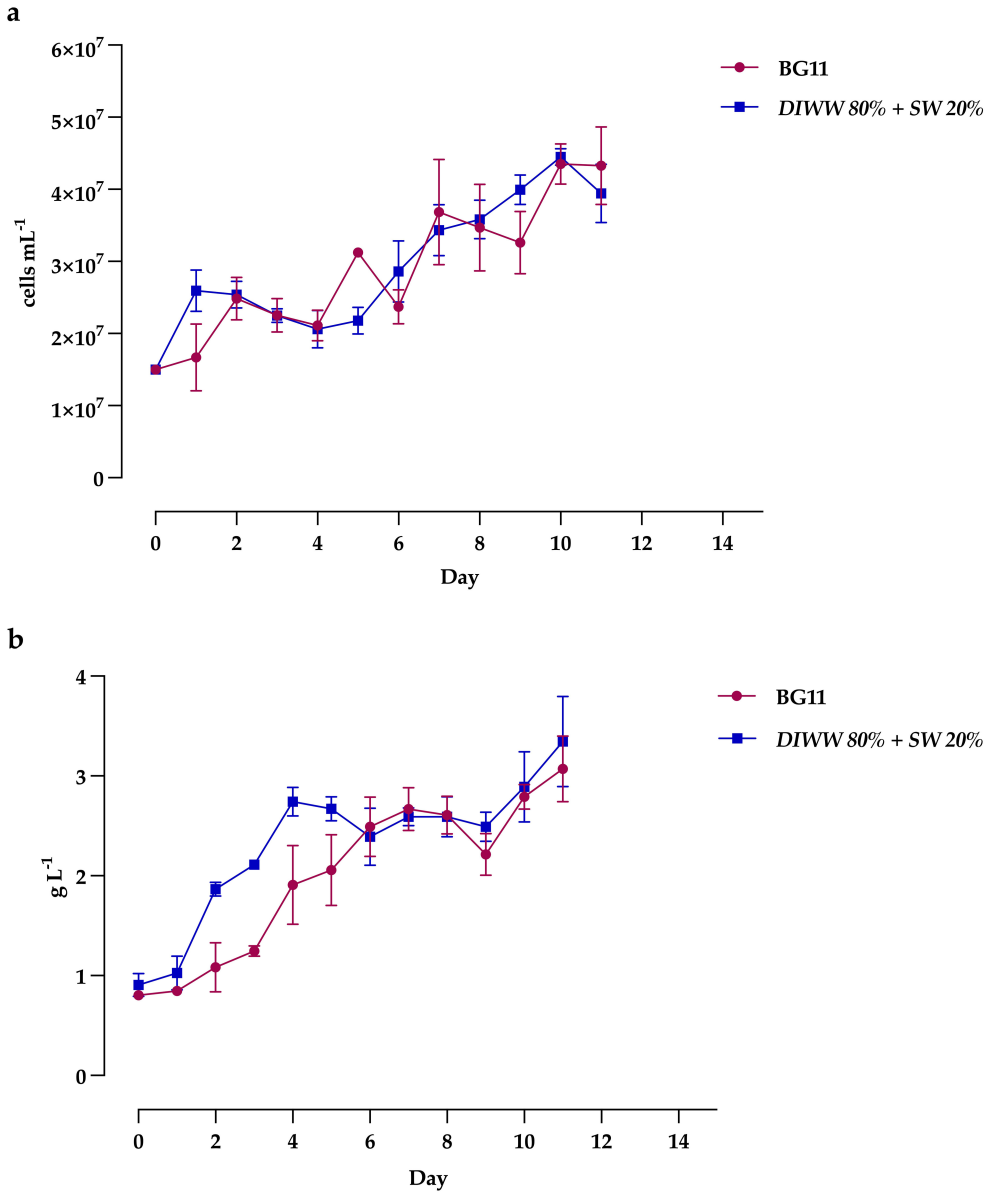
Growth of *Scenedesmus* sp., determined by **(a)** cell density and **(b)** biomass production, cultured in BG11 and DIWW 80% + SW 20% media. Data are presented as the mean ± standard deviation (error bars) of four photobioreactors per treatment, with three independent measurements per replicate.

The growth of *Scenedesmus* sp. on batch cultures is shown in **Figure 3**. Results show that the culture in the wastewater of the dairy industry at 80% showed a higher cell density (4.4 ± 0.1 x 10^7^ cell mL^-1^) compared to the control with the BG11 medium, where a maximum cell concentration of 4.3 ± 0.3 x 10^7^ cell mL^-1^ was reached, both on day 10 of cultivation (**Figure 3a**). However, it does not represent significant differences. It is important to note that between days 2 and 4, for both treatments, an initial short exponential growth phase is observed, followed by a lag phase or growth decrease. Additionally, in the BG11 medium, the behavior shows fluctuations in cell concentration, with brief periods of increase and decrease, despite being a batch culture.

The results of maximum culture biomass concentration, based on daily dry weight data, show a 9% increase in the alternative medium with wastewater (3.35 ± 0.45 g L^−1^) compared to the standard BG11 medium (3.07 ± 0.33 g L^−1^) with biomass productivity of 0.22 ± 0.05 g L^−1^ day^−1^ and 0.21 ± 0.03 g L^−1^ day^−1^, respectively (**Figure 3b**). It is worth noting that, during days 6-9, both treatments show a phase of cell decrease followed by a linear growth phase, which could indicate an atypical trend for this type of culture.

The results of our research, although showing variable patterns between cell density and biomass production, confirmed the ability to use the native strain *Scenedesmus* sp. as a biotreatment mechanism for dairy wastewater due to its growth capacity in this type of substrates, reflected in the increase of up to three times the initial cell density after 11 days and without significant differences with the standard BG11 medium.

### 3.3 Biochemical composition

Microalgae are characterized by their ability to assimilate nutrients from wastewater and incorporate them into their metabolism to produce compounds with high-added value. In our investigation, promising results were obtained regarding the protein concentration of *Scenedesmus* sp., getting 5% more protein content in the *DIWW 80% + SW 20%* medium compared to the standard BG11 medium, with 366.09 ± 57.87 mg g^-1^ and 384.38 ± 29.13 mg g^-1^ of protein, for control and treatment, respectively. In terms of carbohydrates and lipids, there were no significant differences between the culture in the BG11 medium and the culture in the wastewater, showing the metabolic plasticity of *Scenedesmus* sp. to grow in dairy wastewater: the lipid fraction presented 180.24 ± 33.84 mg g^-1^ and 181.59 ± 21.03 mg g^-1^, while the carbohydrate concentration remained at 364.22 ± 31,84 mg g^-1^ and 367.98 ± 38.06 mg g^-1^, for control and treatment, respectively (Tukey’s test *P ≤ 0.05*).

### 3.4 Nutrients removal

**Figure 4:**
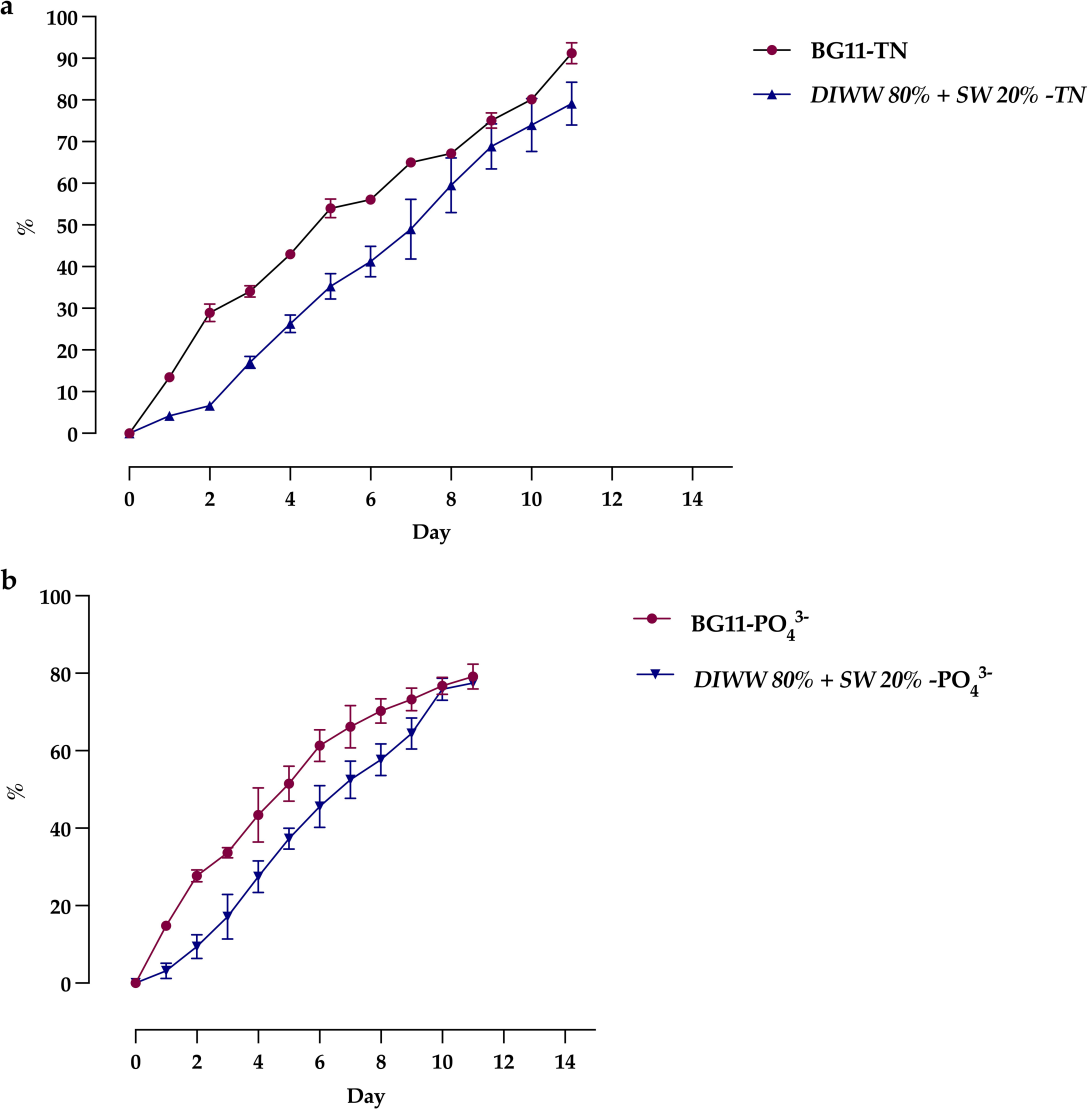
Daily nitrogen **(a)** and phosphorus **(b)** removal by *Scenedesmus* sp. in control (BG11) and treatment media (DIWW 80% + SW 20%). Data are presented as the mean ± standard deviation (error bars) of four photobioreactors per treatment, with three independent measurements per replicate.

Microalgae use the nutrients of the medium as a culture substrate, which is why various low-cost media are being studied for scale production processes. **Figure 4** shows that *Scenedesmus* sp. can take advantage of the nutrients in dairy wastewater, showing, on the 11th day of cultivation, maximum percentages of total nitrogen assimilation of 91.24 ± 3.09% and 79.24 ± 6.26% in the BG11 medium and DIWW 80% + SW 20%, respectively (**Figure 4a**). Regarding total phosphate, percentages of assimilation greater than 75% were observed, with 77.44 ± 1.12% in DIWW 80% + SW 20% and 79.15 ± 3.18% in the BG11 medium (**Figure 4b**).

### 3.5 Vegetative and reproductive development of *Phaseolus vulgaris* L. and soil quality

The pre-sowing analysis of the soil corresponding to the clay loam type showed the following values: CFU g^-1^ 8.63 ± 0.25 x 10^7^, NO_3_^-^ 82.25 ± 0.82 mg g^-1^, PO_4_^3-^ 32.37 ± 0.89 mg g^-1^, SOM 2.33 ± 0.09%, and a pH of 6.15 ± 0.18. The results of the treatments (T_1_, T_2_) revealed a statistically significant difference (Tukey´s test *P ≤ 0.05*) in the concentration of microorganisms (CFU g^-1^) in the two samples concerning the pre-sowing soil sample (S_1_), with the first presenting an average value of 1.27 ± 0.07 x 10^9^ CFU g^-1^ and the second 1.17 ± 0.05 x 10^9^ CFU g^-1^, approximately one order of magnitude higher. No statistically significant differences (Tukey´s test *P* ≤ 0.05) were found in CFU g^-1^ between C- (water) and C+ (fertilizer) concerning the pre-sowing soil count (**Table 4**).

**Table 4:** Physicochemical characterization and quantification of soil CFU g^-1^. Values ​​are averages ± SD (n = 3).

	pH	SOM (%)	NO_3_^-^ (mg kg^-1^)	PO_4_^-3^ (mg kg^-1^)	CFU g^-1^
Pre-sowing soil					
**S_I_**	6.15 ± 0.18	2.33 ± 0.09	82.25 ± 0.82	32.37 ± 0.89	8.63 ± 0.25 x 10^7^
Post-sowing soil					
**C-**	6.05 ± 0.11	2.21 ± 0.08	87.23 ± 3.23	40.40 ± 2.11	8.95 ± 0.57 x 10^7^
**C+**	6.29 ± 0.11	2.33 ± 0.08	73.37 ± 3.43	43.75 ± 2.62	5.19 ± 0.32 x 10^7^
**T_1_**	6.50 ± 0.12	3.47 ± 0.12	107.07 ± 4.08	71.73 ± 1.35	1.27 ± 0.07 x 10^9^
**T_2_**	6.51 ± 0.09	3.23 ± 0.09	94.76 ± 0.76	75.38 ± 3.14	1.17 ± 0.05 x 10^9^

With the parameter NO_3_^-^, statistically significant differences are presented between pre-sowing soil (S_1_) and both treatments (T_1_; T_2_) (Tukey´s test *P ≤ 0.05*) thus the treatment with the microalgae extract presented an increase of 30.18% (107.07 ± 4.08 mg kg^-1^), and the microalgae culture 15.21% (94.76 ± 0.76 mg kg^-1^), the negative and positive control did not present significant differences with S_1_ (Tukey´s test *P ≤ 0.05*), as shown in **Table 4**.

PO_4_^3-^ showed significant differences (Tukey´s test *P ≤ 0.05*) in all plots evaluated concerning S_1_; thus, in the - and + controls, it increased by 24.81 and 35.16% (40.40 ± 2.11 mg kg^-1^; 43.75 ± 2.62 mg kg^-1^), respectively. For T_1_ and T_2,_ this increase was 121.59 and 132.87%, respectively (71.73 ± 1.35 mg kg^-1^; 75.38 ± 3.14 mg kg^-1^) (**Table 4**).

The soil organic matter (SOM) showed the same response as observed with NO^3^-, with no significant differences (Tukey’s test, P ≤ 0.05) between C- and C+ concerning S1; however, T_1_ and T_2_ did show significant differences (Tukey’s test, P ≤ 0.05), with an increase of 48.93 and 38.63%, respectively (3.47 ± 0.12%; 3.23 ± 0.09%), as detailed in **Table 4**.

The soil pH values ​​did not show significant differences (Tukey´s test *P ≤ 0.05*) between the pre-sowing soil and the rest of the plots evaluated (post-sowing soil). Values ​​within the following range were observed: 6.05 - 6.51 (**Table 4**).

**Figure 5:**
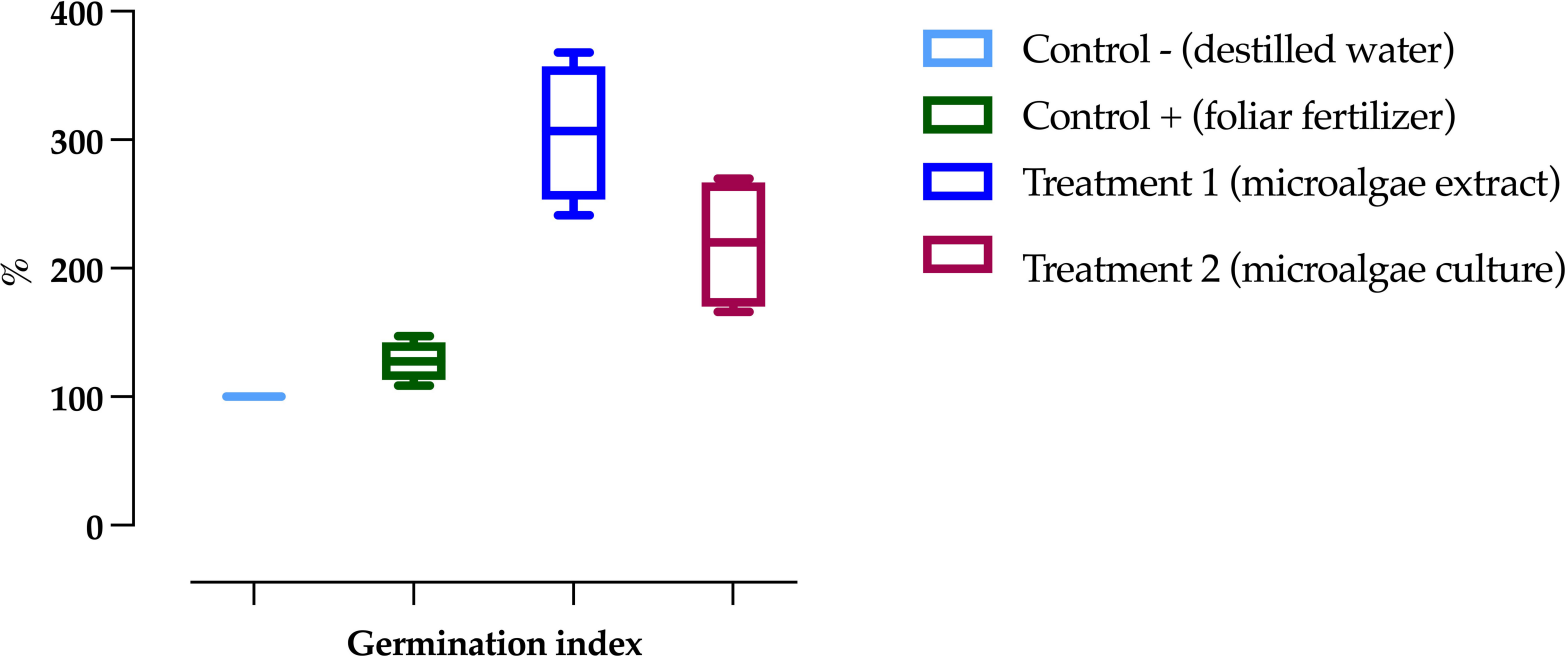
Germination index of common bean (*Phaseolus vulgaris*) seeds under different treatments. Data are presented as the mean ± standard deviation (error bars) of four Petri dishes per treatment, each containing 25 seeds.

The germination assays of *Phaseolus vulgaris* seeds showed the highest indices in T_1_ (305.81 ± 53.62), followed by T_2_ (219.07 ± 52.58), as shown in **Figure 5**. The positive control (C+) exhibited the lowest index, with 127.80 ± 15.67, representing an increase of 2.39 times in T_1_ and 1.71 times in T_2_ compared to C+.

**Figure 6:**
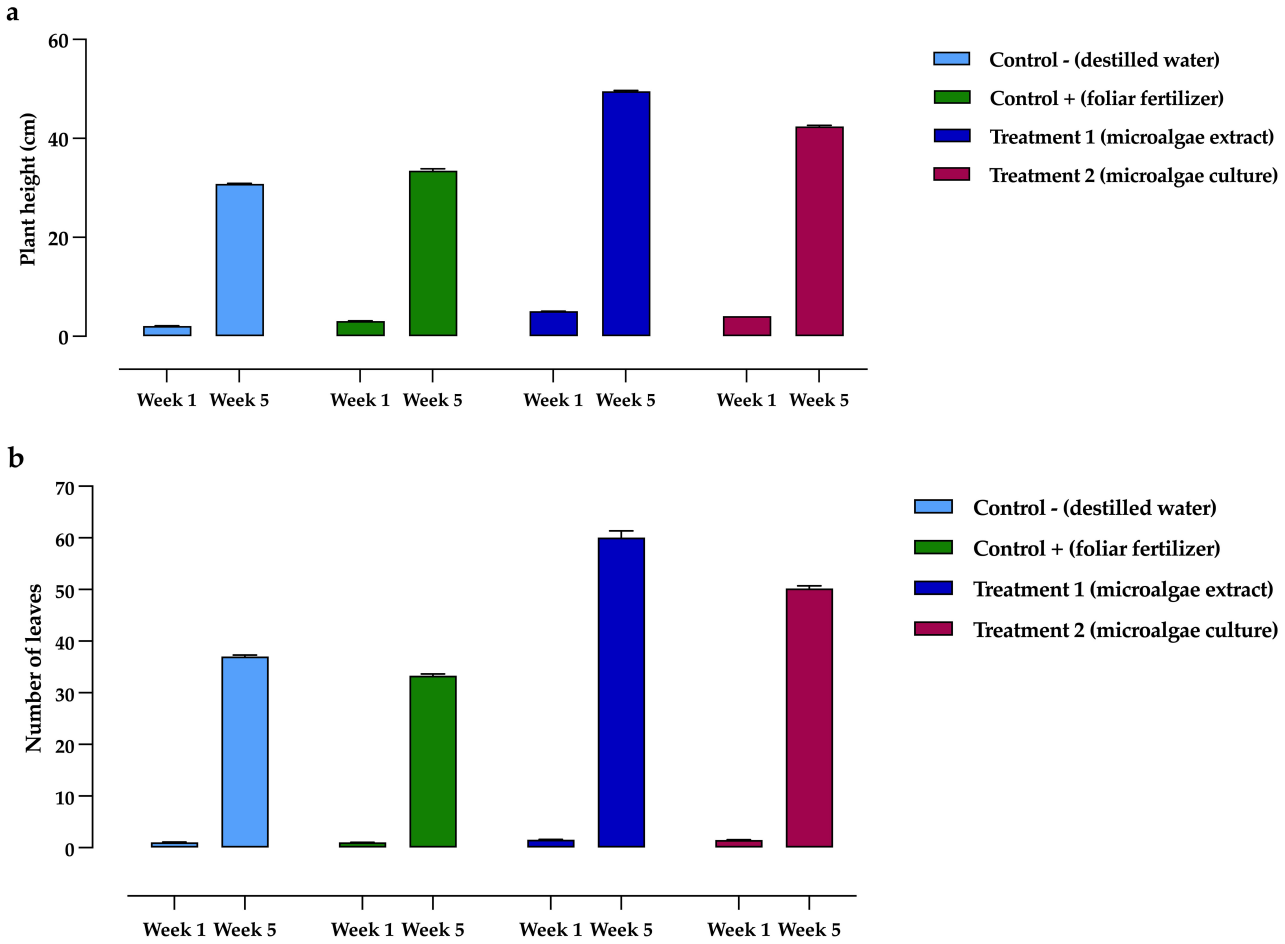
Development of *Phaseolus vulgaris* plants exposed to different treatments, comparing initial (week 1) and final (week 5) measurements. **(a)** Height, **(b)** Leaf development. Data are presented as the mean ± standard deviation (error bars) of four treatments, with three replicates (plots) per treatment, and three repetitions per plot.

Regarding plant height, as shown in **Figure 6a**, significant differences were observed between treatments and controls. In the first week, the highest values were recorded in T_1_ and T_2_, with 5.07 ± 0.34 cm and 4.10 ± 0.19 cm, respectively, while the lowest value was observed in C-, with 2.10 ± 0.15 cm. By the fifth week, T_1_ had achieved the most significant height at 49.52 ± 1.17 cm, whereas C- had the lowest height, measuring 30.82 ± 1.40 cm.

In *P. vulgaris* plants exposed to the treatments, T_1_ showed the highest leaf coverage on average, as seen in **Figure 6b**. In the first week, T_1_ exhibited the highest values (1.55 ± 0.63 leaves), while C+ had the lowest (1.03 ± 0.17 leaves). By the fifth week, the highest leaf counts were recorded for T_1_ and T_2_, with 60.12 ± 2.23 and 50.18 ± 2.02 leaves, respectively, whereas C+ had the lowest, with 33.31 ± 1.50 leaves.

During pre-flowering, where the number of flower buds was determined, T_1_ showed a more significant influence on the reproductive development of *P. vulgaris*, reaching an average quantity of 24.30 ± 1.50 flower buds. At the same time, C+ obtained the lowest value, with 12.15 ± 1.61. Like pre-flowering, during flowering, T_1_ reached the highest values, with an average of 54.58 ± 1.05 developed flowers, followed by T_2_ with 23.81 ± 1.20, while C+ obtained the lowest values, with 15.32 ± 0.91 flowers.

## 4 Discussion

The Scenedesmaceae family comprises diverse taxonomic groups, as in the genus *Scenedesmus*, where 124 species have been accepted (Guiry and Guiry, 2024). This diversity is mainly attributed to its high degree of phenotypic plasticity, making its morphological identification almost invalid. Therefore, applying molecular tools through barcodes for gene analysis or a specific region allows species identification quickly and efficiently. However, no gene ensures 100% of the functionality of an ideal barcode, as it presents a high genetic variability between species (Zou et al., 2016). In the present investigation, the molecular analysis of strain LBM-0020 was performed by PCR amplification and Sanger sequencing with the use of the ITS barcode (primers ITS1 and ITS4), resulting in the analysis of a partial sequence of the small subunit and large rRNA and a complete sequence for the ITS1 region, the 5.8s rRNA gene and ITS2. Bellemain et al. (2010) used various combinations of nuclear DNA ITS for fungal identification of environmental samples, showing that ITS1 and ITS4 regions amplify many sequences for plant species of different orders. However, these ITS regions show significant differentiation in amplifying the sequences, causing mismatches and pairing erroneous sequences that cause an information bias. Sarwa and Verma (2017) studied the similarity of two closely related microalgae corresponding to *Scenedesmus* sp. and *Acutodesmus* sp. through molecular characterization with the amplification of their sequences with the primers ITS1 and ITS4, obtaining a similarity range of 99-97% between the two strains of microalgae, according to the NCBI database.

Cell growth, generally measured as cell concentration in cells mL^−1^, is a key parameter for evaluating the effectiveness of alternative media. In our study, growth in the formulated medium with dairy wastewater and the control medium exhibited a relatively constant pattern, with adaptation, exponential, and stationary phases. However, during days 2-4, a lag phase was observed following an initial exponential phase. BG11 medium, and even non-standard media, although nutrient-rich, may present irregular availability of certain key compounds, such as nitrogen or phosphorus, during the early stages of cultivation. This could lead to a temporary nutrient limitation, affecting cell division but not necessarily biomass accumulation. Previous studies have shown that *Scenedesmus* sp. is capable of accumulating biomass in the form of energy reserves even under nutritional stress conditions, which could explain the absence of growth in the initial days and the discrepancy between cell density and dry weight (Xin et al., 2010; Mandotra et al., 2014). Additionally, it is important to mention that in a batch culture, nutrients are available from the beginning, but their consumption is not uniform. Although the BG11 medium is nutrient-rich, it may exhibit irregular availability of key nutrients, which affects cell division and explains the fluctuations in cell density observed in the cell growth curve. On the other hand, biomass may show a more stable trend because *Scenedesmus* sp. cells can accumulate energy reserves (such as lipids, carbohydrates, or proteins) even when cell division slows down due to temporary nutrient limitations. This explains the possible erratic behavior in cell concentration in the control medium but not in biomass concentration (Hu et al., 2008; Griffiths and Harrison, 2009). Furthermore, the decrease in biomass observed between days six and nine, followed by a new increase from day ten onward, suggests that *Scenedesmus* sp. cells underwent a metabolic adjustment phase in response to changes in the medium conditions. This behavior could be due to a temporary limitation of key macronutrients, prompting the cells to accumulate energy compounds rather than increasing in size or dividing, as previously explained. Subsequently, the increase in biomass from day ten indicates that the cells successfully adapted and resumed growth by utilizing the accumulated reserves, a phenomenon previously reported in microalgae cultivated in batch systems (Griffiths and Harrison, 2009; Xin et al., 2010; Markou and Georgakakis, 2011).

Currently, studies on the *Scenedesmus* genus in dairy effluents that highlight growth advantages use other parameters, such as optical density or biomass concentration. However, research like that of Mamani (Mamani Condori et al., 2024) demonstrated that *Scenedesmus* sp. can grow in raw effluents derived from dairy product production, such as cheese whey. Nevertheless, the authors observed a 1.36-fold reduction in cell density compared to the control (BG11 medium) when using only 10% raw cheese whey diluted in distilled water. This decrease could be attributed to the specific characteristics of the effluent, such as its low nitrogen content, high phosphate concentration, and high turbidity. In another study with dairy wastewater (DWW), Luzzi et al. (2024) analyzed the cultivation of *Chlorella* sp. in dairy wastewater at laboratory and pilot scales in plastic-bags photobioreactors, using different dilutions (1:10, 1:20, 1:30, 1:40), compared to a control medium (BG11-AM6). The results showed that the cell density obtained in all dilutions with wastewater was lower than that of the control at both laboratory and pilot scales. However, at smaller volumes, only the 1:10 treatment showed similar values to the control, with a decrease of 1.04 times compared to the standard medium. At the pilot scale (80 L), the 1:30 treatment showed a decrease of 1.45 times compared to the control. It is important to note that the treatments reached their maximum growth on the third day, while the control reached it by the seventh day. These results may be attributed to the dilution of the initial 35 mM nitrogen to less than 3.5 mM, resulting in very low nutrient levels, which could have slowed down or even inhibited growth due to nutrient depletion over time. Additionally, the authors previously conducted a sedimentation process for the wastewater, which involves additional steps if large-scale production is intended. Similarly, other authors (Hamidian and Zamani, 2022) investigated filtered waste from a dairy plant to culture the *Chlorella sorokiniana* EAKI strain with different dilution rates using the standard BBM medium. The investigation showed that, by day 11, the 100% dilution of DWW resulted in a 5.02-fold increase in cell concentration compared to BBM. These results are favorable and show similar behavior to that observed in the present study. However, the increase reported in their research was significantly higher than ours (2.33-fold), which is attributed to the use of the BBM medium, which provided an advantage by offering a more controlled and nutrient-rich environment compared to our approach, which used seawater as a base.

It has been demonstrated that agro-industrial wastewater represents a viable alternative substrate for producing microalgal biomass for industrial purposes. Our study observed a 9.12% increase in maximum production and a 4.76% increase in productivity with the DIWW 80% + SW 20% treatment, compared to the BG11 medium. These increases are specific to the wastewater characteristics used, which can vary depending on the origin, location, and timing of the effluent’s reception, thus specifically affecting the metabolic processes of each microalgal strain (Hussain and Bhattacharya, 2019). For example, the research by de Mendonca et al. (2022) compared the effectiveness of two microalgal strains in vertical alveolar panel photobioreactors to evaluate their behavior in a medium composed of synthetic dairy product wastewater, simulating the effluents from a cheese factory. They used *S. obliquus* (ACOI 204/07) and *C. vulgaris* in batch and continuous cultures, and observed that the *Scenedesmus* strain exhibited better growth in both types of culture, with a 21.57% and 13.04% increase in the maximum biomass concentration for batch (two days less than *Chlorella*) and continuous cultures, respectively. Additionally, productivity increased by 52.63% (batch) and 73.33% (continuous) compared to the spherical unicellular chlorophyte. Although the high capacity of this *Scenedesmus* strain is demonstrated, it is important to note that the authors implemented a two-phase system, where the first phase involved a pretreatment process using an activated sludge system to reduce substances that could inhibit microalgal growth if the effluent were used as generated. Corresponding to the genus of the Scenedesmaceae family, Mercado et al. (2020), when studying the growth of *Scenedesmus* sp. in 100% dairy wastewater, observed opposite results, as the standard BG11 medium allowed for a greater yield than the dairy effluent, with a 7.14% increase in biomass and a 4.89% increase in productivity. The advantage observed in our study could be because the 20% seawater complemented the formulated medium with additional micronutrients, thus promoting better performance in biomass production.

The use of different nutrient sources can modify the flow of assimilated energy toward the main biomolecules present in microalgal biomass. In the present study, a production of 37%, 38%, and 18% of carbohydrates, proteins, and lipids, respectively, was obtained. The evaluation of different strains allows for analyzing their ability to redirect metabolism toward the accumulation of specific molecules depending on the culture conditions. Ma et al. (2023), using raw dairy wastewater for the cultivation of *Scenedesmus* SDEC-13, *C. pyrenoidosa* SDEC-35, and *C. vulgaris* SDEC-34, observed that all three strains tended to increase their protein and carbohydrate content compared to the BG11 medium. Specifically, *Scenedesmus* SDEC-13 reached 45% protein and 15% carbohydrates, compared to the control, which showed 27% and 11%, respectively. However, the SDEC-34 and SDEC-35 strains exhibited greater accumulation, with a maximum of 50% protein and 25% carbohydrates. Regarding lipid content, all strains presented lower values than those obtained in BG11, with 15% for SDEC-13 and SDEC-35, and 22% for SDEC-34. Compared to our study, Ma et al. (2023) reported a 1.18-fold higher protein content in *Scenedesmus* strains cultivated in dairy wastewater media, while carbohydrate and lipid contents were 2.47 and 1.2 times lower, respectively. Nevertheless, it is important to note that the authors performed a preliminary filtration process using six layers of gauze, which reduced the presence of insoluble solids and other compounds in the medium, potentially influencing the final biomass composition. Nevertheless, this type of pretreatment increases process costs at an industrial scale, which could affect its feasibility for commercial applications. Another study (de Mendonca et al., 2022) also compared the biochemical composition of two chlorophytes grown in dairy wastewater in vertical alveolar panel photobioreactors. In this study, a synthetic medium was evaluated for 12 days, simulating the effluents from a cheese production factory, comparing the cultures of *S. obliquus* (ACOI 204/07) and *C. vulgaris*. The results showed carbohydrate, protein, and lipid percentages of 39%, 35%, and 21%, respectively, for *Scenedesmus*, and 38%, 48%, and 18%, for *Chlorella*. Although the protein content in our study was 8.6% higher in *S. obliquus*, *C. vulgaris* showed a 5.3% increase compared to our results. Furthermore, the carbohydrate content remained in the range of 37-39%, and lipid content was between 18-21%. This highlights the variability in protein fraction, even between strains of the same genus. It is important to note that in our study, we used the wastewater without any prior treatment, while the authors (de Mendonca et al., 2022) employed an activated sludge system in the first stage, which reduced the concentration of certain compounds.

The type of light used in the experiment can also induce higher protein production. Thus, cool white fluorescent LED lights favor higher protein concentrations. For example, Gatamaneni et al. (2020) studied the effects of light quality on physiological and biochemical properties in the cultivation of *C. variabilis* and *S. obliquus* in dairy wastewater. They showed protein percentages more significant than 50% by weight dryness of biomass using cold white fluorescent light, unlike energetic compounds that reached concentrations of less than 35%. This is because white lights generally promote a more significant accumulation of compounds derived from nitrogen metabolism and higher photosynthetic efficiency, growth rate, and electron transport rate. However, this can vary according to the substrate culture and strain (Singh and Singh, 2015). Another crucial parameter when evaluating alternative media is the efficiency of nutrient assimilation, as it helps determine the microalgae’s ability to adapt to environmental conditions. Pandey et al. (2024). analyzed this variable by cultivating *Scenedesmus* sp. ASK22 in an indoor pilot-scale photobioreactor and an outdoor pond photobioreactor for 12 days. The strain, isolated from a dairy effluent treatment plant, was grown under batch conditions using a control medium and a simulated dairy effluent medium. The results showed that nutrient assimilation exceeded 99% in laboratory-scale cultures, while outdoor cultures achieved over 84% for both treatments. Similarly, phosphorus removal in indoor cultures was greater than 81%, while outdoor cultures achieved over 68%, demonstrating comparable behavior in both types of media. On the other hand, Gupta et al. (2024) investigated the potential of mixed wastewater (anaerobically digested dairy wastewater + untreated dairy wastewater) as a cultivation medium for *S. acutus* and found nitrogen removal of 93% and phosphate removal of 70% in batch cultures. This observed behavior is similar to the results obtained in the present study, where nitrogen removal exceeded 79% and phosphorus removal exceeded 77% in our medium formulated with dairy wastewater (DIWW 80% + SW 20%). Consequently, these results highlight the potential of our strain in comparison to other studies. However, further analysis is required to identify factors that could enable nutrient assimilation to exceed 90%.

Despite using wastewater from the same type of industries, these works show that the results will depend on the particular characteristics of the microalgae used, such as adaptation to the substrates and metabolic capacity to assimilate the compounds present. Otherwise, the specific composition of the wastewater to be used must be taken into account due to the presence of organic compounds (carbohydrates, animal fats, oils, surface-active agents, volatile compounds) and inorganic compounds (chlorides, heavy metals, sulfur) that can reduce the ability to metabolize nutrients. Khalaji et al. (2021), for example, observed that when cultivating *C. vulgaris* with an initial density of 2.6 x 10^7^ cells mL^-1^ in dairy wastewater at different concentrations (25%, 50%, 75%), the percentages of removal of nutrients such as nitrogen and phosphorus were less than 50%, due mainly to the presence of oils/fats and low concentrations of NO_3_^-^, NH_4_, and PO_4_^-3^, factors that inhibit adequate nutrient removal. Consequently, producing this type of biomass presents multiple applications, and research indicates positive effects on soil fertility. Increasing the concentration of organic matter in the soil significantly enhances ecosystem fertility (Ammar et al., 2022), which directly impacts the phenological parameters of crop plants. In this context, it has been shown that algal biomass substantially increases the concentration of organic carbon in the soil, improving agricultural productivity and yield (Alvarez et al., 2021). Studies have reported that microalgae such as *C. vulgaris* and *S. quadricauda* increase seed germination in various agrarian cropping systems, such as *Beta vulgaris* (Puglisi et al., 2020) and *C. vulgaris* and *Arthrospira platensis*, improving seed quality, seed germination, yield production, and plant growth parameters in rice and maize plants (Refaay et al., 2021). Applying microalgae biomass can improve soil porosity and aeration, facilitating water circulation and nutrient distribution. This, in turn, promotes greater nutrient bioavailability for a more extended period and serves as a nutrient reservoir that plants can utilize. de Siqueira Castro et al. (2017) applied biomass produced with effluents from the meat processing industry in the cultivation of a grass species, observing a significant improvement in the chemical and biological properties of the soil, such as an increase in N content, organic matter, and cation exchange capacity (CEC). Although primarily referring to cations, CEC is closely related to the bioavailability of elements such as P and N, as it reduces the precipitation of insoluble phosphates and nitrates, promoting greater availability (Jiang et al., 2015). Furthermore, a high CEC is linked to a higher content of SOM, indicating a better capacity for nutrient retention in the soil; this is consistent with the results of the present study, where the biomass of *Scenedesmus* significantly increased organic matter, NO_3_^-^, PO_4_^-3^, and microbial density. About the above, studies such as that of Song et al. (2024) in tomato crops with the Xanthophytes *Tribonema* demonstrate that microalgae-based fertilizers not only increase the density of microorganisms in the soil but also modify the microbial structure, promoting the growth of beneficial bacteria and reducing the presence of pathogens, as corroborated by Song et al. (2022)).

The application of microalgal biomass to seeds before sowing improves seed quality and accelerates the germination process. Various studies have demonstrated the benefits of microalgal extracts in seed germination, as discussed in the review by Chabili et al. (2024). For example, Nezamdoost, Ghahremani, and Ranjbar (Nezamdoost et al., 2024) found that *C. vulgaris* extracts in lettuce crops increased the Germination Index (GI) by 1.25 times compared to the control. Similarly, Ferreira et al. (2019) reported a 2.43-fold increase in wheat and barley seeds treated with *S. obliquus*, while Rupawalla et al. (2022) observed a 2.5-fold increase in spinach seeds treated with *Chlorococcum* and *Scenedesmus*. Navarro-López et al. (2023) noted a 1.2-fold increase in *Scenedesmus almeriensis* in *watercress* seeds, and Viegas, Gouveia, and Gonçalves (Viegas et al., 2021) reported a 2.5-fold increase in wheat seeds treated with *C. vulgaris* extracts. It is worth noting that, in all these studies, the GI reached did not exceed 250%, while in our study, we obtained a GI of 306%. This is explained by the fact that microalgal extracts contain bioactive compounds, such as phytohormones, which are involved in the activation of metabolic processes that enable seeds to transition from dormancy to germination, such as gibberellins (GAs) (Lu and Xu, 2015; Wang et al., 2021). Gibberellins also play a key role in developing characteristics such as shoot length and stem development in plants, particularly in cell division and elongation processes, by stimulating the activity of the apical meristem. Cao et al. (2023) and Ng et al. (2024) corroborate the potential of microalgal extracts applied individually or in combination with PGPR (Plant Growth-Promoting Rhizobacteria) or other types of biomass, which result in a greater than 35% increase in shoot and stem length in herbaceous plants such as tomato, onion, rice, and spinach, during the first 20-50 days in genera like *Chlorella* and *Spirulina*. Reviews of various microalgae suggest that cyanobacteria could generate increases of more than 40% compared to commercial fertilizers, as demonstrated by studies from Chabili et al. (2024), which report increases of 53% and 42% in shoot length of wheat seeds inoculated in a hydroponic system with *Phormidium* and *Synechocystis*. It is worth noting that, in the case of *Scenedesmus* sp., a 66% increase was achieved during the first 7 days and a 48% increase by the fifth week, highlighting the advantages of this strain. Furthermore, it has been analyzed that microalgae can contain over 4700 pg mg^−1^ DW of endogenous gibberellins, with gibberellin 6 (GA_6_) actively involved in vegetative development and plant growth (Stirk et al., 2013).

Regarding leaf development, it has been demonstrated that using microalgal biomass significantly increases leaf area and abundance. In the present study, an 80% increase was observed compared to synthetic fertilizers, highlighting the effectiveness of the microalgal extract as a sustainable alternative. Supraja, Behera, and Balasubramanian (Supraja et al., 2020) achieved a 66% increase in leaf density in tomato plants by applying a mixed culture of *Chlorella*, *Scenedesmus*, *Synechocystis*, and *Spirulina*, while Elarroussi et al. (2016) reported up to a 100% increase in leaf area in tomato and pepper plants through foliar applications of *A. platensis* polysaccharide extract. The effect of these extracts is not limited solely to leaf development but also improves parameters related to relative water content (RWC), especially under drought-stress conditions. Kusvuran (2021) observed that applying *C. vulgaris* in broccoli plants increased the RWC by 61%, associated with greater leaf water release and its effect on regulating cellular turgor. Cytokinins regulate this leaf development, mainly the free bases isopentenyl adenine and zeatin, identified in green algae as part of their endogenous content (Stirk et al., 2002; Romanenko et al., 2016), whose quality and concentration are influenced by light regimes and the availability of carbon sources (Han et al., 2018).

The hormones above are crucial in developing floral buds and flower maturation. This process is complemented by the involvement of auxins (petal opening), ethylene (floral senescence), and abscisic acid (ABA) (floral senescence). On the other hand, gibberellins are essential in forming reproductive structures, as they stimulate cell elongation and floral stem growth. At the same time, cytokinins regulate cell division in floral meristems, promote the transition from vegetative to floral meristems, and contribute to forming structures such as petals and sepals (Barbosa and Dornelas, 2021; Janowska and Andrzejak, 2022). Notably, it has been described that tomato seeds treated with *Chlorella* show a higher transcriptional response, overexpressing oleosin genes that promote more developed pollen compared to treatments with commercial fertilizers (Gitau et al., 2023). This effect, evidenced by *Scenedesmus* sp. achieving a 100% increase in floral bud formation, has also been reported in studies such as that of Dias et al. (2016). Applying a foliar biostimulant based on *Spirulina* in eggplant plants resulted in a 46% increase in floral bud development. Similarly, Rathod et al. (2023) demonstrated that using microalgae in *Cucumis sativus* crops under greenhouse conditions increased floral bud formation by 39%. Additionally, microscopic analysis showed a symbiotic association between the microalgae and the roots, known as “Phyco-rrhiza.” Continuing with floral development, Bayona-Morcillo et al. (2022) reported a 56-59% increase in open flowers when applying *C. vulgaris* hydrolysates to *Pelargonium × hortorum* plants. Similarly, Suchithra et al. (2022) observed a 56% increase in tomato crops using 100% extracts of the same microalgal species. On the other hand, Gitau et al. (2022) achieved a 33% increase in floral development of tomato plants when applying live *Chlorella* sp. cells, significantly reducing the time needed to achieve these results. In the present study, we achieved a 256% increase compared to synthetic fertilizer, demonstrating that the strain used could have a higher concentration of gibberellins and cytokinins while also inducing the biosynthesis of these phytohormones in the crop, as mentioned by Alling et al. (2023).

Finally, the biomass of the native microalga *Scenedesmus* sp. due to its interesting biochemical composition obtained when cultivated in a medium with dairy wastewater, shows great potential for future sustainable applications, such as soil enhancers, biofertilizers, and animal feed, representing more cost-effective production processes.

## 5 Conclusions

The findings of this research demonstrate that the native microalga *Scenedesmus* sp. exhibits favorable growth in dairy wastewater, yielding promising results in terms of growth parameters and biochemical composition of the biomass. Notably, the high protein content of the biomass highlights its potential for applications in agriculture and animal feed. Furthermore, *Scenedesmus* sp. demonstrates a remarkable metabolic capacity to assimilate organic compounds present in wastewater at high rates, making it an exceptional candidate for agro-industrial wastewater biotreatment processes. The application of *Scenedesmus* biomass obtained from this type of wastewater in agriculture has shown significant benefits, including increased germination, plant height, leaf density, number of flower buds, and flower production in legumes such as *Phaseolus vulgaris*, outperforming synthetic chemical fertilizers in these aspects. Looking ahead, future research should focus on optimizing cultivation conditions to further improve biomass productivity and biochemical composition, especially on a large scale. Furthermore, exploring the potential of genetically modified *Scenedesmus* sp. strains could improve their nutrient uptake efficiency and biomass yield. Integrating *Scenedesmus*-based wastewater treatment systems with circular economy principles, such as the production of biofertilizers and high-value bioproducts, could provide sustainable solutions for both environmental management and agricultural productivity. Furthermore, long-term field studies are required to assess the ecological and economic impacts of using *Scenedesmus* biomass in agriculture, thus ensuring its viability as a sustainable alternative to conventional practices. These advances could pave the way for the widespread adoption of microalgae-based technologies in wastewater treatment and sustainable agriculture, thereby contributing to global efforts regarding environmental conservation and food security.

## Data Availability

The datasets presented in this study can be found in online repositories. The names of the repository/repositories and accession number(s) can be found in the article/**Supplementary Material**.
